# Supplementary material for: Stress Granule Induction in Rat Retinas Damaged by Constant LED Light
Source: Invest Ophthalmol Vis Sci. 2025 Jan 15;66(1):38. doi: 10.1167/iovs.66.1.38 (PMC11741064; doi:10.1167/iovs.66.1.38)
Supplement: Supplement 1 [file iovs-66-1-38_s001.pdf]

Supplemental Material 1: Statistical analysis of SG number per cell changes in retinal layers during DR progression (Fig. 4B-C)

a) Combined effect of light exposure duration and retinal layer on SG count per cell

<u>**Scheirer-Ray-Hare test**</u>

**DV**	Number SGs / cell
**Observations**	359
**D**	1.9961031
**MS total**	10711.17

	Df	Sum Sq	H	p
**Light treatment**	4	140824	13.2	0.01034
**Retinal layer**	2	2968178	278.2	0.00000
**Light:Layer**	8	35216	3.301	0.91408
**Residuals**	342	655509		

Dunn`s test with a Bonferroni correction: light treatment (Z and p)

	LD	LL2	LL4	LL6
**LL2**	-3.3693<br>**0.0038**			
**LL4**	-2.3330<br>0.0982	1.1227<br>1.0000		
**LL6**	-2.8090<br>**0.0248**	1.1227<br>1.0000	-0.6196<br>1.0000	
**LL8**	-3.0150<br>**0.0128**	0.3800<br>1.0000	-0.7416<br>1.0000	-0.0881<br>1.0000

Dunn`s test with a Bonferroni correction: retinal layer (Z and p)

	GCL	INL
**INL**	8.0511<br>**0.0000**	
**ONL**	16.6914<br>**0.0000**	8.6395<br>**0.0000**

b) Effect of light treatment on each retinal layer

<u>**Ganglion Cell Layer (GCL)**</u>

ANOVA test followed by Tukey's post hoc test

	Df	Sum Sq	Mean Sq	F value	Pr (>F)
**Light condition**	4	65.73	16.433	9.136	2.11E-06
**Residuals**	109	196.05	1.799		

	LD	LL2	LL4	LL6
**LL2**	1.60E-06			
**LL4**	0.03508	0.03436		
**LL6**	0.00074	0.74849	0.57122	
**LL8**	0.00016	0.80203	0.39887	0.99984

Confidence level used: 0.95

<u>**Inner Nuclear Layer (INL)**</u>

Kruskal-Wallis rank sum test

	Chi-sq	dF	p
**Light condition**	22.84	4	0.000136

Dunn`s test with a Bonferroni correction: light condition (p)

	LD	LL2	LL4	LL6
**LL2**	0.0018			
**LL4**	0.0753	1.0000		
**LL6**	0.0049	1.0000	1.0000	
**LL8**	0.0000	1.0000	0.1409	1.0000

Confidence level used: 0.95

<u>**Outer Nuclear Layer (ONL)**</u>

Kruskal-Wallis rank sum test

	Chi-sq	dF	p
**Light condition**	13.267	4	0.01004

Dunn`s test with a Bonferroni correction: light condition (p)

	LD	LL2	LL4	LL6
**LL2**	0.0128			
**LL4**	1.0000	0.0780		
**LL6**	0.0600	1.0000	0.2727	
**LL8**	0.1680	1.0000	0.6952	1.0000

Confidence level used: 0.95

c) Differential SG counts across retinal layers under different light treatments

Light/Dark cycle (LD)

Kruskal-Wallis rank sum test

	Chi-sq	dF	p
**Retinal layer**	29.291	2	4.36E-07

Dunn`s test with a Bonferroni correction: retinal layer (p)

	GCL	INL
**INL**	0.0569	
**ONL**	0.0000	0.0016

Two days in continuous light (LL2)

Kruskal-Wallis rank sum test

	Chi-sq	dF	p
**Retinal layer**	63.497	2	1.63E-14

Dunn`s test with a Bonferroni correction: retinal layer (p)

	GCL	INL
**INL**	0.0001	
**ONL**	0.0000	0.0030

Four days in continuous light (LL4)

Kruskal-Wallis rank sum test

	Chi-sq	dF	p
**Retinal layer**	69.665	2	7.46E-16

Dunn`s test with a Bonferroni correction: retinal layer (p)

	GCL	INL
**INL**	0.0001	
**ONL**	0.0000	0.0000

Confidence level used: 0.95

Six days in continuous light (LL6)

Kruskal-Wallis rank sum test

	Chi-sq	dF	p
**Retinal layer**	53.645	2	2.24E-12

Dunn`s test with a Bonferroni correction: retinal layer (p)

	GCL	INL
**INL**	0.0001	
**ONL**	0.0000	0.0003

Eight days in continuous light (LL8)

Kruskal-Wallis rank sum test

	Chi-sq	dF	p
**Retinal layer**	66.692	2	3.30E-15

Dunn`s test with a Bonferroni correction: retinal layer (p)

	GCL	INL
**INL**	0.0002	
**ONL**	0.0000	0.0000
