# Supplementary material for: Stress Granule Induction in Rat Retinas Damaged by Constant LED Light
Source: Invest Ophthalmol Vis Sci. 2025 Jan 15;66(1):38. doi: 10.1167/iovs.66.1.38 (PMC11741064; doi:10.1167/iovs.66.1.38)
Supplement: Supplement 2 [file iovs-66-1-38_s002.pdf]

Supplemental Material 2: Normalization by area of the data presented in Figure 4

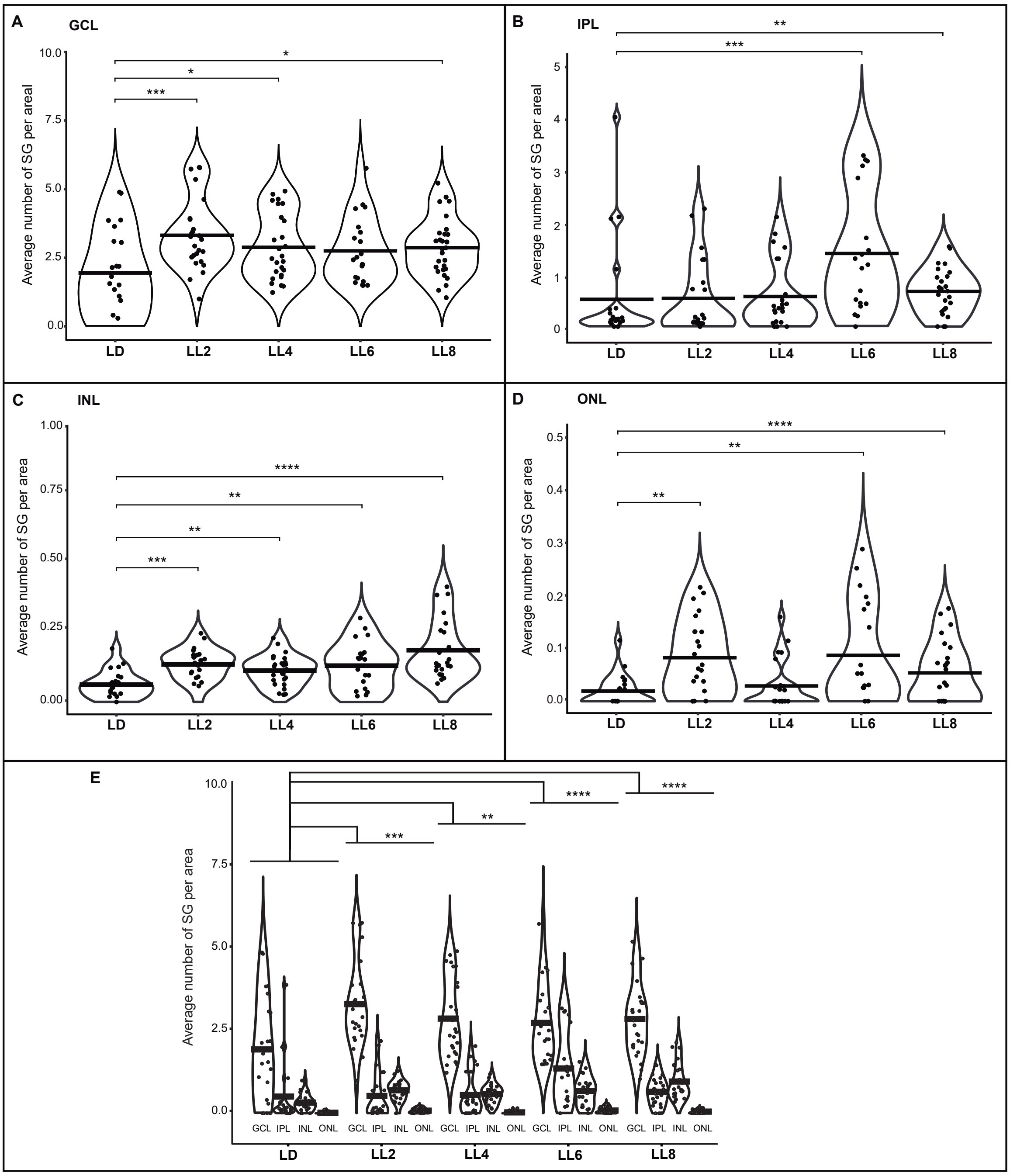

Statistical analysis

a) Combined effect of light exposure duration and retinal layer on SG count per area

Scheirer-Ray-Hare test

**DV**	Number SG/Area
**Observations**	462
**D**	0,99621
**MS total**	17825,5

	Df	Sum Sq	H	p
**Light treatment**	4	336958	18,975	0,00079
**Retinal layer**	3	5243822	295,294	0,00000
**Light:Layer**	12	186934	10,527	0,56985
**Residuals**	442	2407357		

Dunn`s test with a Bonferroni correction: light treatment (Z and p)

	LD	LL2	LL4	LL6
**LL2**	-3,10088<br>**0,00096**			
**LL4**	-2,46831<br>**0,00679**	0,69097<br>0,24479		
**LL6**	-3,79698<br>**0,00007**	-0,81426<br>0,20775	-1,49199<br>0,06785	
**LL8**	-3,79022<br>**0,00008**	-0,63213<br>0,26365	-1,34965<br>0,08856	0,22253<br>0,41195

Dunn`s test with a Bonferroni correction: retinal layer (Z and p)

	GCL	IPL	INL
**IPL**	9,7652<br>**0,0000**		
**INL**	8,2734<br>**0,0000**	1,4641<br>0,4295	
**ONL**	17,1058<br>**0,0000**	7,1814<br>**0,0000**	8,6487<br>**0,0000**

b) Effect of light treatment on each retinal layer

Ganglion Cell Layer (GCL)

Kruskal-Wallis rank sum test

	Chi-sq	dF	p
**Light condition**	22,840	4	0,000054

	LD	LL2	LL4	LL6
**LL2**	0,0004			
**LL4**	0,0178	0,0926		
**LL6**	0,051	0,0522	0,3543	
**LL8**	0,0112	0,1158	0,4389	0,2999

Confidence level used: 0.95

Inner Plexiform Layer (IPL)

Kruskal-Wallis rank sum test

	Chi-sq	dF	p
**Light condition**	17,139	4	0,008170

Dunn`s test with a Bonferroni correction: light condition (p)

	LD	LL2	LL4	LL6
**LL2**	0,2941			
**LL4**	0,1296	0,2826		
**LL6**	0,0001	0,0008		
**LL8**	0,0098	0,0384	0,1129	0,0595

Confidence level used: 0.95

Iner Nuclear Layer (INL)

Kruskal-Wallis rank sum test

	Chi-sq	dF	p
**Light condition**	25,117	4	0,000048

Dunn`s test with a Bonferroni correction: light condition (p)

	LD	LL2	LL4	LL6
**LL2**	0,0001			
**LL4**	0,0065	0,0969		
**LL6**	0,0038	0,1856	0,3681	
**LL8**	0,0000	0,1007	0,0039	0,0156

Confidence level used: 0.95

Outer Nuclear Layer (ONL)

Kruskal-Wallis rank sum test

	Chi-sq	dF	p
**Light condition**	15,121	4	0,00446

Dunn`s test with a Bonferroni correction: light condition (p)

	LD	LL2	LL4	LL6
**LL2**	0,0011			
**LL4**	0,0649	0,0969		
**LL6**	0,0038	0,1856	0,3681	
**LL8**	0,0000	0,1007	0,0039	0,0156

Confidence level used: 0.95

c) Differential SG counts across retinal layers under different light treatments

Light/Dark cycle (LD)

Kruskal-Wallis rank sum test

	Chi-sq	dF	p
**Retinal layer**	35,241	3	1,08E-07

Dunn`s test with a Bonferroni correction: retinal layer (p)

	GCL	IPL	INL
**IPL**	0,0027		
**INL**	0,0223	0,2203	
**ONL**	0,0000	0,0010	0,0001

Confidence level used: 0.95

<u>Two days in continuous light (LL2)</u>

Kruskal-Wallis rank sum test

	Chi-sq	dF	p
**Retinal layer**	66,766	3	2,10E-14

Dunn`s test with a Bonferroni correction: retinal layer (p)

	GCL	IPL	INL
**IPL**	0,0000		
**INL**	0,0000	0,0421	
**ONL**	0,0000	0,0246	0,0001

Confidence level used: 0.95

<u>Four days in continuous light (LL4)</u>

Kruskal-Wallis rank sum test

	Chi-sq	dF	p
**Retinal layer**	74,906	3	3,80E-16

Dunn`s test with a Bonferroni correction: retinal layer (p)

	GCL	IPL	INL
**IPL**	0,0000		
**INL**	0,0000	0,1078	
**ONL**	0,0000	0,0010	0,0000

Confidence level used: 0.95

<u>Six days in continuous light (LL6)</u>

Kruskal-Wallis rank sum test

	Chi-sq	dF	p
**Retinal layer**	56,029	3	4,14E-12

Dunn`s test with a Bonferroni correction: retinal layer (p)

	GCL	IPL	INL
**IPL**	0,0027		
**INL**	0,0000	0,0669	
**ONL**	0,0000	0,0000	0,0014

Confidence level used: 0.95

<u>Eight days in continuous light (LL8)</u>

Kruskal-Wallis rank sum test

	Chi-sq	dF	p
**Retinal layer**	79,935	3	2,20E-16

Dunn`s test with a Bonferroni correction: retinal layer (p)

	GCL	IPL	INL
**IPL**	0,0000		
**INL**	0,0001	0,1135	
**ONL**	0,0000	0,0002	0,0000

Confidence level used: 0.95
